# From multisensory integration to synaesthesia: a boundary-preserving continuum and research framework

**DOI:** 10.3389/fpsyg.2026.1893904

**Published:** 2026-09-23

**Authors:** Kaiyu Qiu, Zixiu Jiang, Ke Jiang, Huilin Qiu

**Affiliations:** 1 College of Humanities, Lishui University, Lishui, China; 2 Shanghai Sivona Education Technology Co., Ltd., Shanghai, China; 3 College of Teacher Education, Lishui University, Lishui, China; 4 College of Foreign Languages and Cultures, Sichuan University, Chengdu, China

**Keywords:** 4E cognition, cross-modal correspondence, education, multisensory integration, neuroplasticity, sensory evolution, symbolic learning, synaesthesia

## Abstract

Synaesthesia is commonly defined as an involuntary and consistent phenomenon in which stimulation in one sensory or cognitive domain elicits an additional experience in another domain. Yet definitions derived from perceptual psychology, neuroscience, aesthetics, cultural anthropology, and artistic practice often speak past one another. This article proposes a boundary-preserving conceptual review that uses the evolutionary history of sensing as a meta-theoretical platform for dialogue among these definitions. It makes four contributions. First, it clarifies the boundaries among congenital or developmental synaesthesia, acquired or trained synaesthesia, synaesthesia-like associations, cross-modal correspondences, multisensory integration, and cultural or metaphorical synaesthesia. Second, it argues that multisensory integration and cross-modal correspondence provide shared cross-modal resources, whereas synaesthesia requires stricter evidence of individual consistency, automaticity, phenomenological vividness, durability, and behavioral interference. Third, it reconstructs the continuum as a heuristic framework rather than a developmental ladder, thereby preserving diagnostic boundaries while enabling communication across competing definitions. Fourth, it explores a dialogue space between synaesthetic artists and psychologists of perception, with implications for scientific research, education, arts-based learning, and cultural psychology.

## Introduction

Synaesthesia is commonly defined as a perceptual phenomenon in which stimulation of one sensory or cognitive pathway, such as seeing letters or hearing sounds, automatically and consistently elicits an additional experience in another sensory or cognitive domain, such as seeing colors, tasting shapes, or experiencing spatial forms (Cytowic, 2002; Ramachandran and Hubbard, 2001). A grapheme-color synaesthete, for example, may experience the letter A as red or the number 5 as green across many years. Such associations are typically idiosyncratic, stable, involuntary, and resistant to intentional modification. These features distinguish synaesthesia from ordinary association, metaphor, memory imagery, and deliberate artistic symbolism.

Historically, synaesthesia was often treated as a rare perceptual curiosity. Early observations, including Galton (1880) study of visualized numerals, attracted scientific interest, but the topic was marginalized during much of the behaviorist period. It returned to sustained scientific attention with the rise of cognitive neuroscience, psychophysics, and neuroimaging. Modern studies have suggested that synaesthesia may be associated with distinctive patterns of structural and functional connectivity, including differences in sensory and parietal regions involved in binding, attention, and conscious perceptual integration (Grossenbacher and Lovelace, 2001; Hubbard et al., 2005; Rouw and Scholte, 2007).

At the same time, contemporary research has complicated the older view that synaesthesia is a fixed and purely innate trait. Training studies have shown that non-synaesthetes can acquire stable color-letter or other cross-modal pairings through repeated association, and some participants report experiences that resemble involuntary synaesthetic concurrents (Colizoli et al., 2012; Bor et al., 2014; Rothen and Meier, 2014). Developmental research has demonstrated that infants and children show systematic cross-modal correspondences before they acquire explicit language, including pitch-brightness relations and sound-shape correspondences (Walker et al., 2010; Wagner and Dobkins, 2011; Maurer et al., 2006; Ozturk et al., 2013). Cultural research has also shown that societies organize ritual, artistic, and symbolic systems through intersensory correspondences (Classen, 1993; Howes, 2011).

A review of the definitions of synaesthesia reveals a further problem: these definitions are not always mutually communicable (Eagleman, 2012). Clinical and perceptual definitions usually emphasize involuntary, consistent, and idiosyncratic inducer–concurrent pairings (Cytowic, 2002; Ramachandran and Hubbard, 2001; Simner, 2012). Cognitive-neuroscientific accounts, by contrast, tend to explain synaesthesia in terms of cross-activation, atypical structural or functional connectivity, disinhibited feedback, or cortical excitability (Grossenbacher and Lovelace, 2001; Hubbard et al., 2005; Rouw and Scholte, 2007; Neufeld et al., 2012; Hupé and Dojat, 2015). Artists and cultural theorists, however, often use synaesthesia to describe cross-sensory symbolism, aesthetic translation, intersensory imagination, and culturally organized forms of lived sensory experience (Classen, 1993; van Campen, 2007; Howes, 2011). The goal of this article is therefore not to replace one definition with another, but to use the evolutionary and developmental history of the senses as a meta-theoretical platform for dialogue. Because multisensory integration is widely regarded as a fundamental and evolutionarily ancient feature of perception, it provides a broader framework within which different definitions of synaesthesia can be compared without being collapsed into one another (Stein and Meredith, 1993; Stein and Stanford, 2008; Stein et al., 2014). On this basis, the article seeks to bridge definitional gaps, establish a boundary-preserving continuum of synaesthesia-related phenomena, and open a more productive dialogue between synaesthetic artists and psychologists of perception. Such a dialogue may both promote scientific research on synaesthesia and clarify the value of synaesthesia-related processes for learning and educational practice.

The article therefore has four aims: to clarify the conceptual boundaries among congenital or developmental synaesthesia, acquired or trained synaesthesia, synaesthesia-like associations, cross-modal correspondences, multisensory integration, and cultural or metaphorical synaesthesia; to develop a boundary-preserving continuum based on diagnostic specificity and evidential demands rather than a linear developmental ladder; to propose a testable framework for studying how cross-modal relations become stable, meaningful, and useful for learning, symbolic development, and artistic cognition; and to foster a more fluent dialogue between synaesthetic artists and psychologists of perception. The central claim is cautious rather than reductive. Synaesthesia should neither be reduced to a neurodevelopmental anomaly nor generalized into all forms of multisensory cognition. Rather, it occupies the most diagnostically specific end of a broader family of cross-modal phenomena, where shared resources for binding, prediction, and meaning extraction become unusually explicit, individualized, automatic, durable, and phenomenologically vivid. In this sense, synaesthesia offers a window into how humans bind sensation, symbol, memory, artistic imagination, and educational practice.

## Conceptual boundaries: synaesthesia and related cross-modal phenomena

A major difficulty in interdisciplinary discussions of synaesthesia is conceptual overextension. The term is often used to describe several different phenomena: genuine congenital synaesthesia, acquired or trained sensory associations, audiovisual illusions, cross-modal correspondences, metaphorical language, and culturally patterned forms of sensory symbolism (Ramachandran and Hubbard, 2001; Grossenbacher and Lovelace, 2001; Spence, 2011; Mroczko-Wasowicz and Werning, 2012). These phenomena are related, but they are not identical. A clearer theoretical account requires distinguishing them before integrating them.

Congenital or developmental synaesthesia refers to the most widely studied form of synaesthesia. It usually emerges early in life, persists over time, and involves automatic and consistent inducer-concurrent pairings. The inducer may be a grapheme, sound, word, odor, taste, pain, spatial sequence, or conceptual category; the concurrent may be color, spatial form, texture, taste, movement, affective tone, or other sensory or quasi-sensory experience. Synaesthetes are often divided into projectors and associators: projectors experience the concurrent as externally located, whereas associators experience it as an internal but vivid mental association (Ramachandran and Hubbard, 2001).

Trained synaesthesia-like associations refer to pairings acquired through deliberate and repetitive training. These effects may show some features of synaesthesia, including consistency, partial automaticity, and interference effects (Colizoli et al., 2012; Bor et al., 2014; Rothen and Meier, 2014). However, researchers who adopt stricter definitions would regard such outcomes do not automatically qualify as congenital or developmental synaesthesia. If a trained association were to satisfy all five criteria proposed below–automaticity, consistency, phenomenological vividness, durability, and behavioral interference–it should not be dismissed merely because it was acquired. It would be more precise to distinguish it from congenital synaesthesia by origin and developmental timing, while reserving the term synaesthesia-like association for cases that satisfy only partial criteria (Baron-Cohen et al., 1987; Eagleman et al., 2007; Simner, 2012; Rothen and Meier, 2014; Bor et al., 2014; Colizoli et al., 2014; Meier, 2015; Rothen et al., 2018; Schwartzman et al., 2023).

Cross-modal correspondences are systematic associations shared by many people, such as the tendency to pair high pitch with bright colors, sharp shapes with high tones, rounded pseudowords with rounded shapes, or large objects with low sounds (Spence, 2011; Maurer et al., 2006; Ozturk et al., 2013; Fort et al., 2015; Chow and Ciaramitaro, 2019). Here, population-level means that the mapping is statistically shared across many individuals rather than idiosyncratic to one person. Developmental-level means that the mapping can be observed in infancy or childhood before full linguistic or explicit symbolic mediation. These correspondences can facilitate perception, communication, and learning, but they do not necessarily involve the involuntary concurrent experiences characteristic of synaesthesia.

Multisensory integration refers to the neural and behavioral process by which information from different senses is combined into a coherent percept (Shams and Beierholm, 2010). The ventriloquist effect and the McGurk effect are classic examples. In the ventriloquist effect, visual location can bias perceived sound location; in the McGurk effect, visual speech cues alter auditory speech perception (Alais and Burr, 2004; McGurk and MacDonald, 1976). These effects demonstrate that perception is inherently constructive and multisensory, but they are not synaesthesia in the strict sense because they do not involve stable, idiosyncratic inducer-concurrent mappings.

Cultural or metaphorical synaesthesia refers to intersensory mappings organized through language, ritual, art, and symbolic practice. Expressions such as “warm color,” “sharp sound,” or “heavy tone” reflect embodied and culturally learned mappings between sensory domains (Classen, 1993; Howes, 2011; Lakoff and Johnson, 1999; Lacey et al., 2012). These forms of synaesthesia are important for understanding art, religion, metaphor, and education, but they should be treated as cultural-symbolic phenomena unless they are accompanied by the perceptual features of individual synaesthesia.

Table 1 summarizes the major distinctions among these phenomena. The table is not intended to draw rigid borders; rather, it identifies diagnostic criteria that prevent the concept of synaesthesia from becoming too broad to be theoretically useful. The most important distinctions concern whether the mapping is individual or shared, automatic or strategic, perceptually vivid or symbolic, stable or context-dependent, and idiosyncratic or semantically motivated.

**Table 1:** Diagnostic distinctions among synaesthesia and related cross-modal phenomena.

Phenomenon	Defining features	Relation to synaesthesia	Evidential caution
Multisensory integration	Combination of inputs from different senses into a coherent percept.	A general perceptual mechanism that may support cross-modal binding.	Not synaesthesia unless it produces stable, idiosyncratic inducer-concurrent mappings.
Cross-modal correspondence	Population-level or developmental tendency to associate features across modalities.	A shared probabilistic basis for learning, communication, and symbolic scaffolding.	Can be early and robust, but usually does not involve individual, involuntary concurrents.
Cultural/metaphorical synaesthesia	Intersensory mappings shaped by language, ritual, art, and symbolic practice.	A cultural-symbolic use of sensory relations.	Should not be treated as individual synaesthesia without perceptual evidence.
Trained/acquired synaesthesia-like association	Pairings acquired through deliberate practice, repeated exposure, or culturally structured training.	May show consistency, partial automaticity, vividness, and interference; in rare cases it may approach acquired synaesthesia.	Requires evidence of vividness, involuntariness, durability, behavioral consequences, and control for attention or memory strategies.
Congenital/developmental synaesthesia	Early-emerging, stable, idiosyncratic, automatic inducer-concurrent pairing.	The strict and most widely studied form of synaesthesia.	Requires consistency, subjective vividness, automaticity, and long-term stability.

The table distinguishes phenomena by diagnostic criteria rather than by rigid categorical boundaries.

The conceptual boundaries and mutual relationships among these phenomena are illustrated in Figure 1. The figure should be read as a boundary-preserving heuristic continuum, not as a taxonomic hierarchy or developmental ladder. At one end are ordinary multisensory integration and shared cross-modal correspondences; in the middle are culturally learned, artistically elaborated, and training-induced synaesthesia-like mappings; at the other end are congenital, developmental, acquired, or otherwise highly stable synaesthetic experiences. The value of this continuum is that it preserves the specificity of synaesthesia while allowing it to illuminate broader mechanisms of multisensory cognition, artistic practice, and symbolic learning.

**Figure 1:**
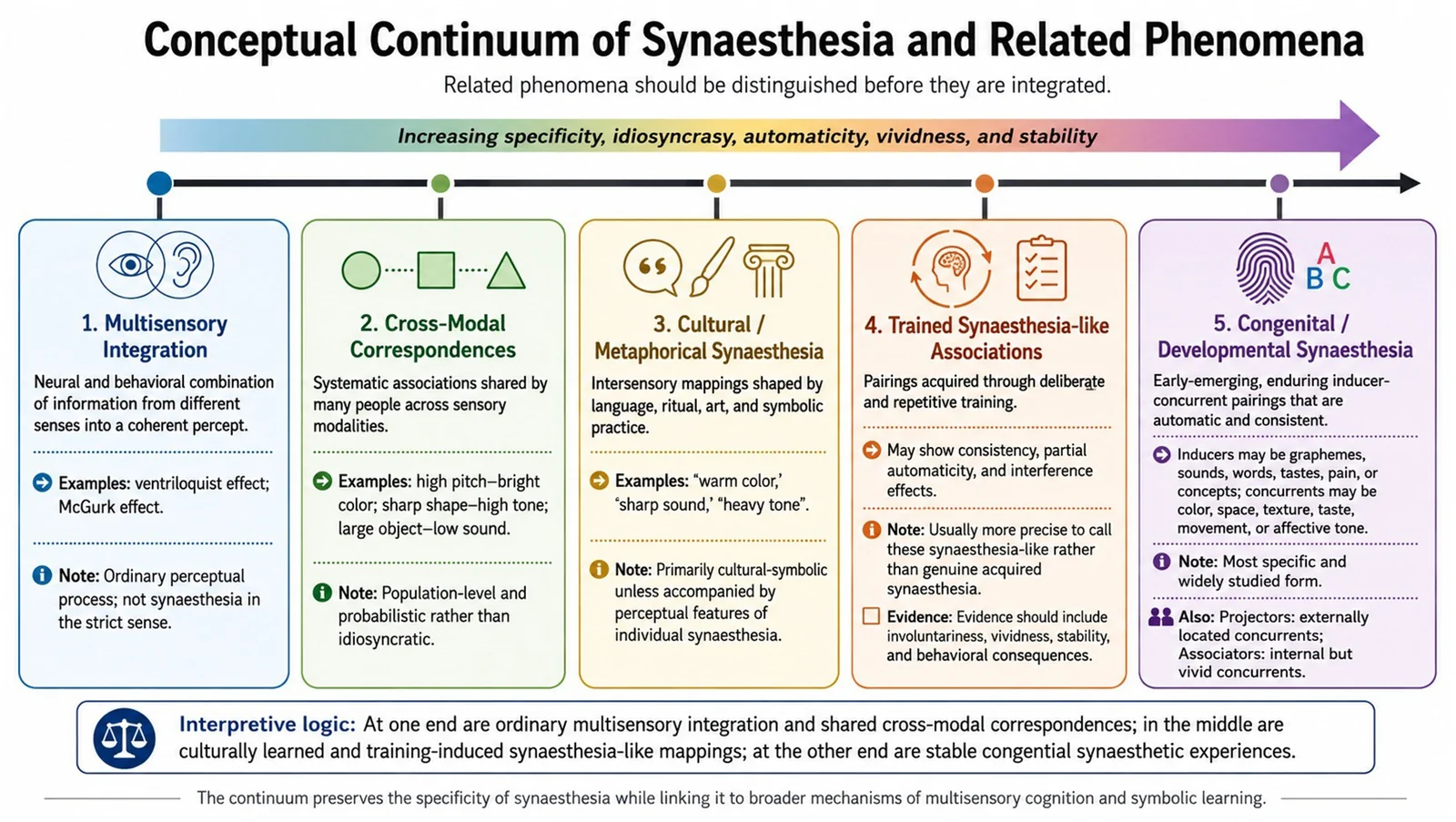
Boundary-preserving continuum of synaesthesia and related phenomena. The continuum ranges from ordinary multisensory integration and shared cross-modal correspondences to cultural/metaphorical and trained synaesthesia-like mappings and, at the most diagnostically specific end, congenital, developmental, acquired, or highly stable synaesthesia. The arrow indicates increasing evidential demands rather than a necessary developmental progression.

## Evolutionary continuity and ontogenetic foundations of multisensory integration and synaesthesia

The evolutionary argument for synaesthesia must be formulated carefully. Current evidence does not directly show that synaesthesia itself evolved as a adaptation in the same sense as vision, audition, or olfaction. The stronger and more defensible claim is that multisensory integration is evolutionarily ancient, and that synaesthesia may reflect how some cross-modal resources can become unusually explicit, individualized, and stable under particular developmental, neurobiological, experiential, or cultural conditions.

From an evolutionary perspective, perception is not a passive recording of the world. It is an active interface through which organisms identify nutrients, avoid danger, locate mates, coordinate movement, and regulate action under uncertainty. Even simple organisms respond to gradients of light, chemicals, vibration, and temperature. As nervous systems became more complex, sensory channels became increasingly differentiated, but they did not become isolated. Adaptive behavior depends not only on sensory specialization but also on the coordination of information across modalities (Keeley, 2002; Stein and Stanford, 2008).

Animal perception illustrates this principle. Bats use echolocation to transform auditory information into spatial representations. Describing this sensory mechanism, however, does not by itself capture the subjective character of the animal's experience (Nagel, 1974). Owls use specialized auditory localization mechanisms to map sound onto space when hunting in darkness (Knudsen and Konishi, 1979). Birds and insects rely on tight visuomotor coupling, while amphibians integrate auditory and visual signals in orienting and prey capture (Wilczynski and Capranica, 1984; Wylie et al., 2009). These examples should not be described as synaesthesia in the strict human sense. Although these examples do not directly establish the evolutionary origin of human synaesthesia in the strict sense, they suggest that cross-modal linkage among sensory experiences has adaptive value in biological evolution.

To understand how this evolutionary architecture manifests in individuals, one must also examine ontogenetic foundations. Human development provides an important line of evidence (Lickliter and Bahrick, 2004). Infants show sensitivity to cross-modal correspondences before they possess mature language or explicit symbolic knowledge. They can match auditory pitch with visual features such as size or brightness (Walker et al., 2010), and developmental work on sound-shape correspondence shows that the bouba-kiki effect can appear early in life while also changing across development (Maurer et al., 2006; Ozturk et al., 2013; Chow and Ciaramitaro, 2019). Wagner and Dobkins (2011) further reported that shape information modulated color preferences in 2- and 3-month-old infants but not in 8-month-olds or adults, a finding directly relevant to the neonatal synaesthesia hypothesis.

On the basis of such evidence for early cross-modal perceptual sensitivity in infancy, Maurer (1993) proposed the neonatal synaesthesia hypothesis. According to this hypothesis, infants may initially experience a transiently less differentiated sensory world because of dense and insufficiently pruned cortical connectivity. As neural pruning and functional differentiation proceed during normal development, this early synaesthetic-like state is gradually reduced or disappears, leaving most cross-modal correspondences in a more implicit and flexible form. From this viewpoint, congenital synaesthesia may be understood as the atypical retention or stabilization of early cross-modal connectivity. Although the neonatal synaesthesia hypothesis remains influential, it is still one developmental account among others rather than a settled explanation. Its value lies in supporting the plausibility of developmental continuity between early cross-modal sensitivity and later synaesthetic experience, but it does not justify treating all infant cross-modal correspondences as early synaesthesia.

Neurally, multisensory integration is supported by structures and networks such as the superior colliculus, posterior parietal cortex, intraparietal sulcus, and distributed cortical association areas (Stein and Meredith, 1993; Stein and Stanford, 2008). These systems support the real-time coordination of perception and action. In humans, regions such as the intraparietal sulcus have also been implicated in some forms of synaesthesia, suggesting a possible overlap between ordinary binding mechanisms and unusual cross-modal experience (Hubbard et al., 2005).

Taken together, evolutionary and neurodevelopmental evidence suggests a possible view: synaesthesia reflects latent possibilities within a brain architecture designed for multisensory prediction, binding, and meaning extraction. In this sense, synaesthesia is not an error in perception, but a distinctive stabilization of cross-modal relations that are normally more flexible, implicit, shared, and context-dependent.

## A boundary-preserving continuum: shared resources without category collapse

Figure 1 presents a boundary-preserving continuum of synaesthesia and related cross-modal phenomena. The model arranges five forms of cross-modal experience according to increasing diagnostic specificity and evidential demands: multisensory integration, cross-modal correspondence, cultural or metaphorical synaesthesia, trained or acquired synaesthesia-like mappings, and congenital or developmental synaesthesia. At the left end, multisensory integration refers to the general neural and behavioral capacity to combine information across sensory channels into coherent percepts. Cross-modal correspondence refers to shared probabilistic mappings, such as pitch–brightness or sound–shape associations, that are often early, robust, and population-level rather than idiosyncratic. Cultural or metaphorical synaesthesia occupies a middle position because it organizes intersensory meaning through language, ritual, art, and symbolic practice. Trained or acquired synaesthesia-like mappings involve associations strengthened by training, attention, culture, or altered states, but they require tests of vividness, automaticity, durability, and interference before stronger claims can be made. Congenital or developmental synaesthesia occupies the most diagnostically specific end of the continuum because it involves early-emerging or highly stable inducer–concurrent mappings that are idiosyncratic, involuntary, consistent, and phenomenologically vivid.

This boundary-preserving logic also helps bridge the gap between synaesthetic artists and psychologists of perception. For example, artists often explore cross-sensory translation, affective resonance, and symbolic correspondence, whereas psychologists require operational evidence of consistency, automaticity, vividness, durability, and interference. The continuum allows these two perspectives to communicate without being reduced to one another. Artistic practice can generate hypotheses about cross-modal salience and symbolic transformation, while psychological research can test which forms of cross-modal mapping become automatic, durable, and educationally useful. In this sense, the model offers not only a conceptual clarification of synaesthesia-related phenomena, but also a shared platform for scientific, artistic, and educational inquiry.

This model may face an immediate objection: if these phenomena are placed on a continuum, does the framework not risk collapsing the very distinctions it seeks to preserve? This is a serious concern, and it is precisely why the continuum must not be read as a linear developmental ladder. The model does not claim that multisensory integration naturally develops into cross-modal correspondence, that cross-modal correspondence naturally becomes synaesthesia-like representation, or that synaesthesia is merely an intensified version of ordinary perception. Rather, the continuum functions as a comparative framework. It identifies shared cross-modal resources while preserving diagnostic boundaries among different phenomena. The arrow in Figure 1 therefore indicates increasing diagnostic specificity and evidential demands, not increasing complexity, value, adaptiveness, or developmental maturity.

In this framework, stabilization is not a metaphorical shortcut for an unproven conclusion. It refers to an operational convergence of several criteria: long-term consistency, automaticity, phenomenological vividness, durability, and behavioral interference. Multisensory integration provides the general capacity for cross-sensory binding; cross-modal correspondence provides shared probabilistic mappings; cultural and artistic practices can make such mappings symbolically salient; and training can strengthen associative automaticity. However, the term synaesthesia becomes warranted only when cross-modal relations converge in an individual, durable, involuntary, and vivid inducer–concurrent relation. Thus, the model does not dissolve synaesthesia into ordinary multisensory cognition; instead, it specifies the evidential threshold at which synaesthesia can be distinguished from weaker or more general cross-modal phenomena.

## Trained, acquired, and temporary synaesthesia-like associations and cross-modal neuroplasticity

If synaesthesia is linked to broader mechanisms of cross-modal plasticity, it becomes reasonable to ask whether synaesthesia-like experiences can be cultivated. Training studies provide evidence that non-synaesthetes can acquire consistent inducer-concurrent pairings, especially in grapheme-color tasks. Participants repeatedly exposed to letter-color associations may later show improved memory for pairings, increased automaticity, and in some cases subjective reports of color experiences when viewing letters (Colizoli et al., 2012; Bor et al., 2014; Rothen and Meier, 2014).

The significance of these findings should not be overstated. Training studies do not prove that congenital synaesthesia can be fully created in adulthood. They show instead that some components of synaesthetic processing–consistency, automatic retrieval, cross-modal association, and partial perceptualization–can be strengthened through learning. If all diagnostic criteria are met, the appropriate contrast is not between real and unreal synaesthesia, but between congenital/developmental synaesthesia and acquired/trained synaesthesia. If only some criteria are met, the more cautious term remains synaesthesia-like association.

A rigorous account of trainability therefore requires explicit operational criteria (Table 2). First, the association should be consistent over time, not merely remembered during the task. Second, the response should show some degree of automaticity, such as interference when the trained concurrent conflicts with the stimulus. Third, subjective reports should be assessed cautiously and combined where possible with behavioral or neural measures. Fourth, durability should be evaluated after training ends, because temporary learning does not establish enduring synaesthetic experience. Fifth, the content of training should be distinguished from ordinary mnemonic strategy, imagery, or voluntary visualization. These criteria also make it possible to evaluate acquired synaesthesia without denying its potential validity in advance.

**Table 2:** Operational criteria for distinguishing synaesthesia, synaesthesia-like association, and cross-modal correspondence.

Diagnostic dimension	Operational indicator	Recommended method
Consistency	Whether the inducer-concurrent pairing remains stable over time	Test-retest consistency task
Automaticity	Whether the concurrent is activated without strategic intention	Synaesthetic Stroop, priming, or congruency task
Phenomenological vividness	Whether the concurrent is experienced as vivid, sensory, or quasi-sensory	Structured interview and vividness rating
Durability	Whether the association persists after training or exposure ends	One-month, three-month, and six-month follow-up
Behavioral interference	Whether incongruent pairings slow or disrupt performance	Congruent/incongruent reaction-time design
Neural plausibility	Whether perceptual, parietal, or connectivity measures change systematically	fMRI, EEG/ERP, DTI, or TMS, where appropriate
Control condition	Whether effects exceed attention, motivation, memory, or practice effects	Active control group and preregistered contrasts

fMRI, functional magnetic resonance imaging; EEG, electroencephalography; ERP, event-related potential; DTI, diffusion tensor imaging; TMS, transcranial magnetic stimulation.

The neural mechanism most often invoked is Hebbian learning: repeated co-activation of neural populations can strengthen functional links between them (Hebb, 1949; Brown, 2020; Langille, 2018). Cross-modal training may therefore recruit association areas and sensory cortices in ways that make formerly independent stimuli more tightly linked (Shams and Seitz, 2008; Proulx et al., 2014; Merabet et al., 2008). Research on sensory substitution and cross-modal plasticity supports this general possibility. For example, in blind or deaf individuals, cortical areas deprived of their usual input can be recruited by remaining senses, showing that sensory boundaries remain plastic across development and, to a limited extent, in adulthood (Bavelier and Neville, 2002; Proulx et al., 2014). Importantly, cross-modal plasticity is not limited to long-term sensory deprivation: temporary blindfolding in normally sighted adults can rapidly and reversibly recruit early visual cortex for tactile processing (Merabet et al., 2008).

However, training effects are not equally likely for all pairings. Semantically or ecologically congruent pairings appear to be easier to learn than arbitrary mappings. High pitch and brightness, rough texture and sharp shape, or heaviness and low sound may be more readily internalized because they align with existing cross-modal regularities (Marks, 1978; Spence, 2011). Attention, imagery, absorption, and motivation may also influence whether a learned association becomes vivid and automatic. Thus, training does not simply impose artificial links on a passive brain; it works by amplifying patterns that are already supported by perceptual, semantic, or cultural structure.

Cross-modal training may improve certain forms of memory, engagement, or symbolic encoding (Shams and Seitz, 2008; Rothen et al., 2012). It may also enrich artistic and language learning by helping learners connect sound, color, movement, affect, and spatial form (Imai et al., 2008; Ozturk et al., 2013; Macedonia and Knösche, 2011; Spence, 2011). Yet the claim that training can reliably enhance creativity or intelligence remains insufficiently demonstrated. Future studies should compare different training protocols, include active control groups, measure long-term durability, and separate subjective reports from behavioral performance.

## Temporary and drug-induced synaesthesia: state-like cross-modal coupling

Temporary and drug-induced synaesthesia provides another important line of evidence. Reviews of chemical induction suggest that psychoactive substances can elicit synaesthesia-like concurrents, often in association with broader changes in attention, affect, imagery, and cortical excitability (Luke and Terhune, 2013). Comparative work also indicates that drug-induced synaesthesia should be distinguished from genuine developmental synaesthesia because it is typically transient, pharmacologically induced, and embedded in altered states of consciousness (Sinke et al., 2012).

This literature is useful for the present framework because it shows that synaesthesia-like experiences need not be treated as mere anomalies. They can arise when neurochemical, attentional, or contextual conditions temporarily alter cross-modal coupling. At the same time, their transient and state-dependent character confirms the need for diagnostic boundaries: temporary drug-induced experiences may reveal possible neural substrates of cross-sensory concurrence, but they do not by themselves establish congenital or acquired synaesthesia unless they satisfy the same standards of consistency, vividness, automaticity, durability, and interference.

## From cross-modal mapping to symbolic learning: the arbitrary-semantic transition

The cognitive importance of synaesthesia and synaesthesia-like processes lies not only in unusual perception but also in their possible contribution to symbolic learning. Human cognition depends heavily on the ability to map relations across domains. Language, mathematics, music, and visual art all require the transformation of sensory experience into abstract structure. Cross-modal mappings may therefore function as scaffolds for meaning, especially when learners encounter symbolic systems that are otherwise arbitrary or difficult to internalize.

In ordinary cognition, cross-modal correspondences can serve as cognitive shortcuts that constrain perceptual binding and facilitate the organization of sensory information (Spence, 2011; Parise and Spence, 2012). For example, high-pitched sounds are commonly associated with visual features such as brightness, small size, and high spatial position, whereas low-pitched sounds tend to be associated with darkness, larger size, and lower spatial position (Evans and Treisman, 2010; Spence, 2011; Walker et al., 2010; Jonas et al., 2017). Learners may therefore use such correspondences to organize perception, memory, and symbolic encoding. Such mappings reduce complexity by binding different features into coherent patterns. This does not mean that synaesthesia is necessary for learning, but it suggests that synaesthetic or synaesthesia-like strategies may enrich encoding by making abstract material more sensorily differentiated.

Language provides a clear example. Many abstract concepts are structured through embodied metaphors: time is understood as movement, emotion as temperature or verticality, and quantity as spatial extension (Lakoff and Johnson, 1999; Lakoff and Núñez, 2000). These metaphors are not identical to synaesthesia, but they show that conceptual thought often relies on cross-domain mapping. Neurocognitive evidence also suggests that processing sensory metaphors can recruit sensorimotor regions, supporting the idea that abstract meaning remains partly grounded in bodily and perceptual systems (Lacey et al., 2012).

The bouba-kiki effect offers a particularly relevant case for the arbitrary-semantic transition. Rounded pseudowords are often matched with rounded shapes, whereas sharp-sounding pseudowords are matched with angular shapes. This sound-shape correspondence has been reported in toddlers and adults (Maurer et al., 2006), in 4-month-old infants (Ozturk et al., 2013), and in later developmental studies showing that the perceptual features prioritized in sound-shape correspondence change with age (Chow and Ciaramitaro, 2019). Such findings show that apparently arbitrary linguistic forms can be partially constrained by cross-modal structure, providing a bridge between sensory evolution, language development, and symbolic learning.

A central theoretical problem is the tension between the idiosyncratic arbitrariness of congenital synaesthesia and the semantic congruence of ordinary cross-modal mapping. In congenital synaesthesia, inducer-concurrent pairings are often non-semantic and arbitrary from a public point of view. Why a particular grapheme evokes a particular shade of red may be stable for the individual but lacks an obvious ecological rationale. In contrast, linguistic metaphors and population-level cross-modal correspondences, such as pairing high pitch with brightness or smallness, are often grounded in environmental regularities, statistical learning, bodily experience, or shared semantic spaces.

This tension is not a contradiction but a transition along the proposed boundary-preserving continuum. Congenital synaesthesia represents a highly individualized stabilization of inducer-concurrent pairing. Cross-modal correspondence represents a shared and probabilistic mapping grounded in perceptual regularities, statistical learning, bodily experience, or early developmental biases. Cultural, artistic, and trained synaesthesia-like representations occupy the middle zone: they can transform arbitrary symbolic materials into meaningful, memorable, and phenomenologically salient structures by aligning them with existing sensory, motor, affective, or semantic relations.

This arbitrary-semantic transition is especially important for education. When a learner uses color-coded notation to master musical pitch, spatial diagrams to understand grammar, or gesture and rhythm to internalize vocabulary, the learner is not necessarily becoming synaesthetic. Rather, the learner is using cross-modal structure to make an arbitrary symbolic system more perceptually organized and semantically accessible. The educational value of synaesthesia-like processing lies precisely in this capacity to translate abstract symbols into multimodal forms that can be perceived, felt, rehearsed, and remembered.

Memory research offers more direct evidence of possible benefits. Some synaesthetes show enhanced memory for materials related to their synaesthetic associations, especially when those associations provide additional cues for encoding and retrieval (Rothen et al., 2012). However, the benefit is domain-specific rather than general. A grapheme-color synaesthete may remember letters, words, or numbers more efficiently because each item has additional color structure, but this does not necessarily imply superior memory across all tasks. Educational applications should therefore focus on specific learning contexts rather than claiming a general cognitive advantage.

Creativity is another promising but complex domain. Synaesthetic experience can support unusual associations, vivid imagery, and cross-domain expression, all of which are relevant to artistic creativity. Poets, painters, composers, and designers often use synaesthetic metaphors because they allow one sensory logic to reorganize another. Yet it would be too strong to conclude that synaesthesia automatically increases creativity. A more precise hypothesis is that stable or trained cross-modal mappings may support particular components of creativity, such as divergent association, multimodal imagery, affective salience, and symbolic recombination.

The most useful theoretical move is therefore to connect synaesthesia-like processing to specific mechanisms: additional encoding cues, cross-domain analogy, affective salience, attentional engagement, and multimodal representation. These mechanisms can be tested empirically. For example, one could examine whether color-sound training improves music memory, whether movement-form mapping supports vocabulary learning, or whether cross-modal art exercises increase originality in constrained creative tasks. Such studies would move the field beyond broad claims about creativity toward measurable cognitive processes.

## Cultural synaesthesia, 4E cognition, and Vygotskian mediation

Synaesthetic perception is not only a neurocognitive issue. It is also shaped by culture, language, art, and education. Many societies do not treat the senses as sharply separated channels. Instead, they organize perception through culturally meaningful relations among sound, color, smell, taste, touch, rhythm, and social order. Howes (2011) used the term cultural synaesthesia to describe such socially shared intersensory systems. Classen (1993) similarly showed that cultures transmit not only knowledge about the world but also preferred ways of sensing and interpreting it. Winkelman (2004) similarly interprets shamanic practices in relation to neuropsychological processes and altered states of consciousness. These cultural systems do not replace perceptual psychology; rather, they identify historically organized materials through which psychologists can formulate testable questions about cross-modal learning.

This cultural stabilization of intersensory associations resonates with contemporary cognitive frameworks, particularly 4E cognition and Vygotsky's socio-cultural historical theory. From the perspective of 4E cognition, the human mind is not an isolated processor manipulating abstract symbols in a vacuum; cognition is embodied in the sensorimotor system, embedded in cultural environments, enacted through practical activity, and extended through tools and media (Mroczko-Wasowicz and Werning, 2012; Newen et al., 2018). Cultural synaesthesia illustrates how artistic, linguistic, and ritual tools can structure how individuals notice, value, and use cross-sensory relations.

When seen through a Vygotskian lens, cultural intersensory practices function as psychological tools (Vygotsky, 1978) that mediate higher mental functions. Just as language reorganizes memory and attention, culturally patterned sensory mappings–such as the deliberate synthesis of color, sound, spatial layout, and movement in rituals, calligraphy, or multimedia art–can reorganize biological multisensory integration into structured symbolic perception. Human synaesthetic potential should therefore be understood not only as a matter of neural connectivity but also as a developmental space shaped by socio-cultural history.

Modern art offers another important case. Kandinsky's reflections on color and sound, Scriabin's experiments with music and colored light, and later multimedia or interactive virtual reality installations all show how artists deliberately explore cross-sensory correspondences (Kandinsky, 1977; Peacock, 1985; Dann, 1998; van Campen, 2007). These practices are not merely decorative. They function as experiments in perception, asking viewers or listeners to experience symbolic relations among sensory domains. Nevertheless, artistic synaesthesia should be discussed carefully. Unless supported by psychological evidence, claims about an artist's actual synaesthetic experience should be distinguished from artistic use of synaesthetic metaphor. This distinction is not intended to deny the value of artists' personal experiences, but to strengthen the scientific rigor of empirical research.

Education may be one of the most promising areas for applied research. Children often learn through multimodal activity: they chant numbers, draw letters, move through space, associate rhythm with language, and use color to organize categories. These practices may not train genuine synaesthesia, but they can cultivate cross-modal sensitivity. In music education, linking pitch, gesture, and visual form may support auditory discrimination. In language learning, connecting phonetic contrast with mouth shape, rhythm, movement, or visual imagery may strengthen audiovisual integration. In art education, translating sound into line, color into movement, or texture into rhythm may help learners develop flexible symbolic expression.

At the same time, educational claims should remain evidence-based. It is plausible that cross-modal training can support memory, attention, and creativity in specific contexts, but it is not yet established that such training produces stable synaesthesia or broad cognitive improvement. The most responsible approach is to treat synaesthetic pedagogy as a set of multisensory learning strategies grounded in cross-modal correspondence and embodied cognition. Its value lies less in making learners become synaesthetes than in helping them build richer links among perception, symbol, emotion, and action.

This reframing also avoids a common misunderstanding. Synaesthesia should not be romanticized as a superior mode of consciousness, nor should non-synaesthetic perception be treated as deficient. Different learners may benefit from different sensory routes. The educational significance of synaesthesia lies in what it reveals about the plasticity of symbolic learning: cognition becomes more flexible when abstract material can be represented through multiple sensory and motor formats.

## Operational criteria and future research agenda

The preceding review suggests the need for a model that is both integrative and empirically testable. Figure 1 provides the boundary-preserving continuum: it distinguishes related phenomena according to diagnostic specificity and evidential demands. Figure 2 translates this continuum into a research framework. The first level is multisensory integration, the ordinary neural process through which the brain combines sensory inputs into coherent percepts. The second level is cross-modal correspondence, in which features across modalities are systematically associated at the population or developmental level. The third level is synaesthesia-like representation, in which training, attention, art, education, or culture stabilizes certain cross-modal pairings and makes them more vivid, automatic, or symbolically meaningful. The fourth level is congenital, acquired, or otherwise highly stable synaesthesia, where inducer-concurrent pairings are idiosyncratic, involuntary, consistent, durable, and phenomenologically vivid.

**Figure 2:**
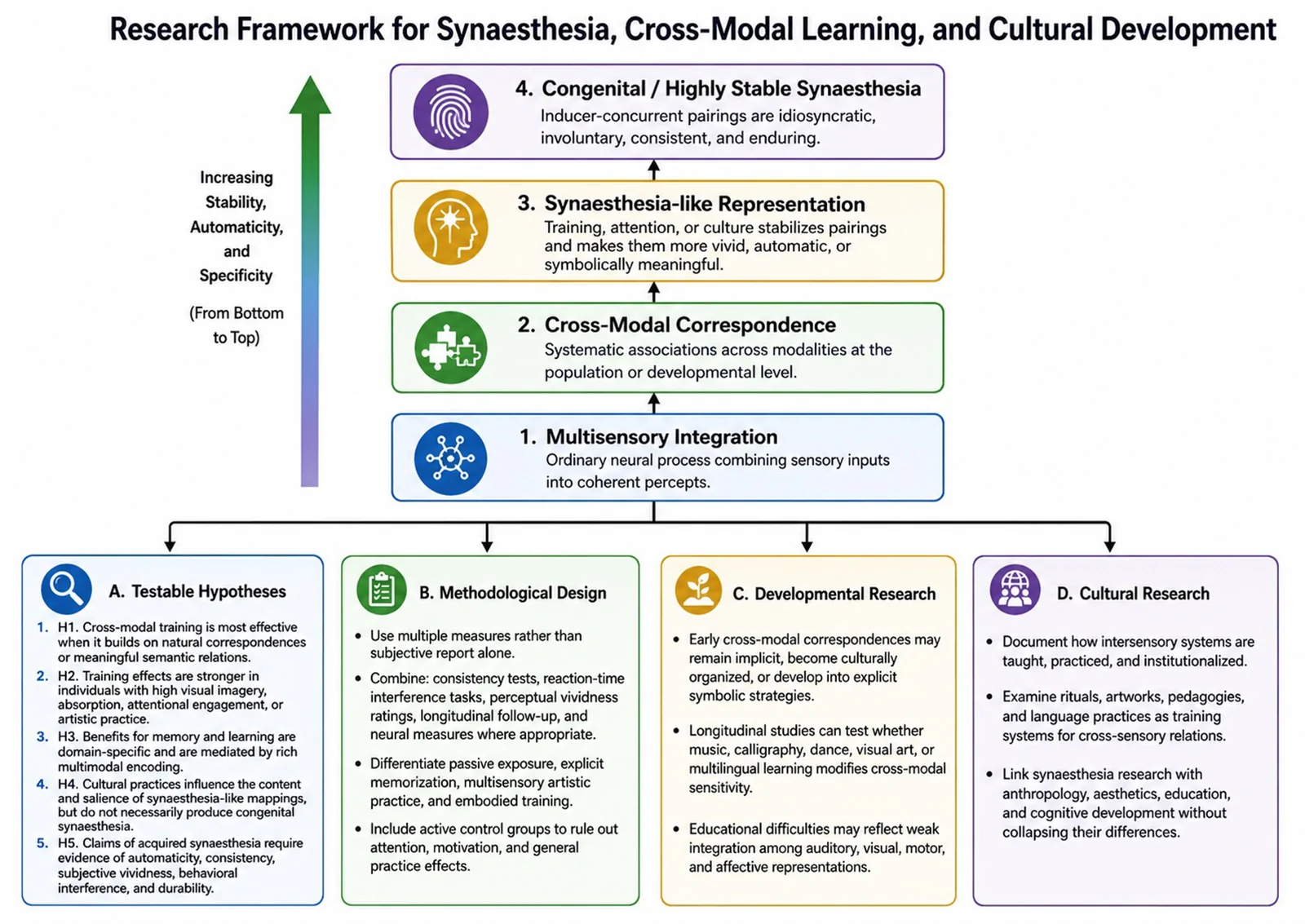
Research framework for synaesthesia, cross-modal learning, and cultural development. The framework uses the evolutionary history of sensing as a meta-theoretical platform and organizes the continuum into research levels, operational criteria, developmental questions, cultural inquiry, and educational applications.

This model makes it possible to formulate more precise hypotheses. First, cross-modal training should be most effective when it builds on natural correspondences or meaningful semantic relations. Second, training effects should be stronger in individuals with high visual imagery, absorption, attentional engagement, or artistic practice. Third, benefits for memory and learning should be domain-specific and mediated by the richness of multimodal encoding. Fourth, cultural practices should influence the content and salience of synaesthesia-like mappings, but not necessarily produce congenital synaesthesia. Fifth, claims of acquired synaesthesia should require evidence of automaticity, consistency, subjective vividness, behavioral interference, and durability. Sixth, artistic reports of cross-sensory experience should be treated as valuable phenomenological materials that can generate hypotheses, but not as substitutes for behavioral or neural evidence.

Methodologically, future research should avoid relying on subjective report alone. A stronger design would combine consistency tests, reaction-time interference tasks such as synaesthetic Stroop paradigms, perceptual vividness ratings, longitudinal follow-up, and neural measures where appropriate. Studies should also distinguish among passive exposure, explicit memorization, multisensory artistic practice, embodied training, and temporary altered states, because these methods may produce different kinds of cross-modal learning. Active control groups are essential; without them, improvements may reflect attention, motivation, cultural familiarity, or general practice rather than synaesthesia-like transformation.

Developmental research should test how early cross-modal correspondences become implicit, culturally organized, or explicit symbolic strategies. Longitudinal studies should examine whether music, calligraphy, dance, visual art, or multilingual learning modifies cross-modal sensitivity, and whether there exists a sensitive period in childhood during which multisensory training is more readily converted into highly automatic synaesthesia-like representations. Such studies would be especially valuable for understanding symbolic learning, because many educational difficulties may arise not from lack of intelligence but from weak integration among auditory, visual, motor, and affective representations.

Environmental and cultural research should document how intersensory systems are taught, practiced, and institutionalized. Rather than treating cultural synaesthesia as metaphor only, researchers can examine how rituals, artworks, pedagogies, and language practices train people to notice and use cross-sensory relations. This would connect synaesthesia research with anthropology, aesthetics, education, and cognitive development without collapsing their differences. It would also provide a more concrete dialogue space in which synaesthetic artists can help psychologists design ecologically valid materials for perception and learning studies.

## Conclusion

Synaesthesia is best understood neither as a mere neurological curiosity nor as a universal label for all cross-sensory experience. It occupies a specific position within a broader, boundary-preserving continuum of multisensory cognition. Ordinary perception depends on multisensory integration; development and culture organize cross-modal correspondences; training, art, education, and altered states can strengthen synaesthesia-like associations; and in some individuals, these relations become stable, involuntary, durable, and phenomenologically vivid as synaesthetic experience.

The strongest current evidence supports the evolutionary continuity and developmental plasticity of multisensory integration. Synaesthesia may be an intensified or stabilized manifestation of these general resources, rather than a direct adaptation in itself. Its educational and artistic significance lies in revealing how perception can become symbolic, how memory can become multimodal, and how creativity can emerge from the translation of experience across sensory domains. The evolutionary history of sensing therefore provides a meta-theoretical platform on which scientific definitions, artistic experience, cultural symbolism, and educational practice can communicate.

For this reason, the study of synaesthesia should move beyond the opposition between innate rarity and acquired skill. The most urgent empirical questions concern whether trained associations can meet the same diagnostic criteria as congenital synaesthesia, whether there are developmental windows for stabilizing cross-modal mappings, and which educational or artistic interventions produce domain-specific benefits without overclaiming broad cognitive enhancement. Such research can clarify the boundaries of synaesthesia while expanding our understanding of human symbolic intelligence, embodied cognition, aesthetic imagination, and creative development.
